# Characterization of *Pseudomonas aeruginosa *isolated from chronically infected children with cystic fibrosis in India

**DOI:** 10.1186/1471-2180-5-43

**Published:** 2005-07-21

**Authors:** Gunjan Agarwal, Arti Kapil, Susheel Kumar Kabra, Bimal Kumar Das, Sada Nand Dwivedi

**Affiliations:** 1 Department of Microbiology, All India Institute of Medical Sciences, Ansari Nagar, New Delhi-110029, INDIA; 2 Department of Pediatrics, All India Institute of Medical Sciences, Ansari Nagar, New Delhi-110029, INDIA; 3 Department of Biostatistics, All India Institute of Medical Sciences, Ansari Nagar, New Delhi-110029, INDIA

## Abstract

**Background:**

*Pseudomonas aeruginosa *is the leading cause of morbidity and mortality in patients with cystic fibrosis (CF). With chronicity of infection, the organism resides as a biofilm, shows multi-drug resistance, diversifies its colony morphology and becomes auxotrophic. The patients have been found to be colonized with multiple genotypes. The present work was carried out to characterize *P. aeruginosa *isolated from children with cystic fibrosis using phenotypic and genotypic methods.

**Results:**

We studied 56 patients with CF attending the Pediatric Chest clinic at All India Institute of Medical Sciences, New Delhi, India during August 1998-August 2001. These patients were regularly followed up at the clinic. Out of 56 patients, 27 were culture positive for *P. aeruginosa *where 8 were chronically infected (Group1) and 19 were intermittently colonized with the organism (Group2). Patients under Group1 had significantly higher rates of hospitalization, death and colonization with different colony morphotypes (p < 0.05). The isolates from Group1 patients were the positive producers of extended spectrum beta lactamase. A total of 5 auxotrophs were recovered from 2 patients where one was chronically infected with *P. aeruginosa *and the other was a recently enrolled patient. The auxotrophs had the specific requirement for methionine and arginine. Molecular typing revealed 33 ERIC-PCR (E1-E33) and 5 PCR-ribotyping (P1-P5) patterns. By ERIC-PCR, 4 patients were colonized with 2–4 genotypes and the remaining 23 patients were colonized with the single genotype.

**Conclusion:**

With chronicity of infection, *P. aeruginosa *becomes multidrug resistant, diversifies its colony morphology, acquires mucoidity and shows auxotrophy for amino acids. The chronically infected patients can be colonized with multiple genotypes. Thus in a particular clinical set up, high index of suspicion should be there for diagnosis of CF patients so as to prevent the delay in diagnosis and management of CF patients.

## Background

Cystic fibrosis (CF) is the recessive genetic disorder of Caucasian race. CF in Asians and Indians was thought to be almost nonexistent in the past. However, recent reports have suggested CF to be more common in Indian population than previously thought. For proper management, it is important to diagnose the infection in the patients with CF [1-3].

The mutations in the cystic fibrosis transmembrane regulator gene in CF patients lead to recurrent and chronic respiratory tract infections, which serves as a major cause of morbidity and mortality [4]. If appropriate antibiotics are used, the quality of life improves in CF patients. Thus, the knowledge of the etiological agents and their antimicrobial susceptibility can help in planning the appropriate antibiotic therapy to be instituted in CF patients.

Infection due to *Pseudomonas aeruginosa (P. aeruginosa) *has been implicated as a major cause of morbidity and mortality in patients with CF [5-8]. Once *P. aeruginosa *colonizes the respiratory passages, it is rarely possible to eradicate it. Prolonged and repeated administration of drugs lead to the development of multidrug resistance in *P. aeruginosa *strains. To detect emergence of the resistant strains, it is important to monitor the antibiotic susceptibility pattern of *P. aeruginosa *isolates. Increased resistance to gentamicin, quinolone and cephalosporins has been reported in chronically infected patients [9,10].

With severity of pulmonary infection, the organism undergoes phenotypic modification, which helps in its persistence under adverse environment of the respiratory tract. The organism diversifies into different colony morphotypes [11,12] and develops antibiotic resistance for its survival. This may be due to an adaptation of the organism to the host defense mechanisms and antibiotic selective pressure [13]. Under recurrent and chronic endobronchial *P. aeruginosa *infections, the initial colonization is with nonmucoid forms of an organism but with progressive lung tissue damage, it gets converted to the mucoid form. The mucoidity helps an organism to grow as biofilm, which serves as a protective niche and helps to evade the host immune response and action of antibiotics. The organism also becomes auxotrophic for amino acid due to high amino acid concentration in the respiratory passages. The most commonly required amino acid reported in literature is methionine while other amino acids-leucine, arginine and ornithine have been required by a minority of *P. aeruginosa *strains [14].

During the course of infection, the organism may undergo genotypic changes. The chronically infected patients have been found to have long-term colonization by one predominant genotype but variation in genotypes within a single patient has also been observed [13,15].

Since no data is available on the characterization of *P. aeruginosa *from CF patients in India, the present study was undertaken to study phenotypic and genotypic variations of *P. aeruginosa *isolated from CF patients attending the Pediatric Chest Clinic at All India Institute Of Medical Sciences (AIIMS), New Delhi, INDIA.

## Results

### Subjects

A total of 56 patients with CF regularly attended the Pediatric Chest Clinic between August 1998-August 2001. These patients were followed monthly at the chest clinic. At the time of enrollment, all the patients were below 15 yrs of age (mean age (yrs) ± standard deviation was 5.33 ± 4.3 and median age was 4.5). The male: female ratio was 2:1.

Out of 56 patients, culture positive infection was observed in 33(58.9%) patients of whom 27(81.8%) were culture positive for *P. aeruginosa *and 6(18.2%) patients were culture positive for organism other than *P. aeruginosa *where one patient each was colonized with *Citrobacter freundii, Staphylococcus aureus, Escherchia coli, Enterobacter *sp and two patients were colonized with *Klebsiella pneumoniae*. The rate of infection with *P. aeruginosa *was significantly higher than the rate of infection with organism other than *P. aeruginosa *in CF patients (p < 0.05). Out of 27 culture positive patients, 8 had chronic infection with the organism and were included in Group1. The remaining 19 patients were intermittently colonized with the organism and were included in Group2.

During the study period, 9 patients required hospitalization and 4 have died. Of the 4 deaths, 3 occurred during the hospitalization. All the patients who required hospitalization were culture positive for *P. aeruginosa *and were followed daily. The mean duration of hospitalization was 12 days (range was 7–20 days). Out of 9 patients, 6 were included in Group1 and 3 in Group 2. All 4 patients who died, had pulmonary exacerbations and were chronically infected with *P. aeruginosa*. The mortality related to *P. aeruginosa *infection in the study was 14.8%. The frequency of hospitalization and death in chronically infected patients was significantly higher as compared to intermittently colonized patients with *P. aeruginosa *(p < 0.05).

### Antimicrobial susceptibility

Three hundred and fifty *P. aeruginosa *isolates were recovered from 27 patients where 94(26.9%) showed the mucoid morphotype (Type5) and the rest 256 (73.1%) isolates showed nonmucoid morphotype(Type1–4 and Type 6). The antimicrobial susceptibility was determined by agar dilution method based on NCCLS guidelines and antibiograms were further constructed (Table 1). One hundred and forty three (41%) isolates were multidrug resistant and showed A3 and A5-A8 antibiogram. All the isolates were recovered from chronically infected patients.

**Figure 1:**
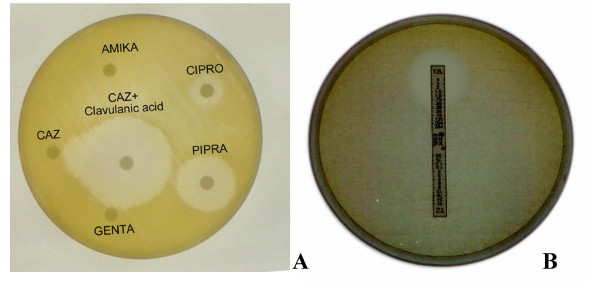
**Detection of Extended spectrum β lactamase in *P. aeruginosa *isolate from a patient (180/95) by:A. Disc Diffusion method **(Amika: Amikacin; Cipro: Ciprofloxacin; Pipra: Piperacillin; Genta: Gentamicin; CAZ: Ceftazidime). **B. Etest method **(AB Biodisk, Sweden).

We further characterized all 29 *P. aeruginosa *isolates that showed resistance to ceftazidime and were the positive producers of extended spectrum beta lactamase (ESBL). These isolates were detected as ESBL positive by disk diffusion according to NCCLS guidelines (Fig 1A) and further confirmation was done by Etest method (Fig. 1B). Twenty-four of the 29 (82.8%) ESBL positive isolates were multidrug resistant (A6 antibiogram). All 29 isolates were found sensitive to imipenam and meropenam.

The ESBL positive isolates were recovered from 3 patients (PCC.No. 180/95, 160/98 and 499/00) where 2 (PCC.No. 180/95 and 160/98) were chronically infected and one patient (PCC.No.499/00) was intermittently colonized. Out of 29 ESBL positive isolates, 27 were recovered from the patient (PCC.No. 180/95) during his different follow-ups past two years and 1 isolate each was recovered from 2 patients (PCC.No. 160/98 and 499/00).

### Phenotypic characterization of *P. aeruginosa *isolates

#### Colony morphotypes

Out of 27 patients infected with *P. aeruginosa*, 7 harbored different colony morphotypes in a single sputum sample while the rest 20 patients harbored Type 1 colony morphotype. All the 7 patients were chronically infected with the organism(Group 1). The rate of colonization with different colony morphotypes of *P. aeruginosa *was significantly higher in Group 1 patients (p < 0.05).

Six (85.7%) of 7 patients harbored mucoid *P. aeruginosa *in their respiratory samples and all were chronically infected. The rate of colonization with mucoid *P. aeruginosa *was significantly higher (75%) in chronically infected patients (p < 0.05). Of the 6 patients who were positive for mucoid *P. aeruginosa*, 3 have died.

Out of 350 *P. aeruginosa *isolates, 194(55.4%) showed Type 1 morphotype. The distribution of antibiogram among colony morphotypes is shown in Table 2.

#### Auxotrophy

Out of 350 *P. aeruginosa *isolates, 5 were auxotrophs, which did not grow on minimal agar medium (MAM) and required Mueller Hinton agar (MHA) for their growth. Of the 5 auxotrophs, four (isolate No.2–5) were isolated from the chronically infected patient (PCC.No.180/95) who was diagnosed as CF six years ago. One auxotroph (isolate No.1) was isolated from a recently enrolled patient (PCC.No.499/00). The respiratory samples collected during their subsequent follow-ups were found negative for the presence of auxotrophs. These auxotrophs were recovered when these patients presented with pulmonary exacerbation.

**Figure 2:**
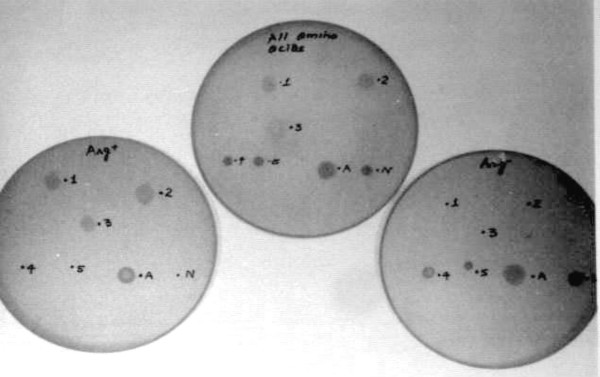
**Detection of Auxotrophy of *P. aeruginosa*. Isolate No: Patient PCC.No. 1: **PCC.No. 499/2000; **2–5: **PCC.No. 180/95, **A: *P. aeruginosa ***ATCC 27853; **N: *P. aeruginosa ***NCTC 50184(meth^-^). **Plate A: **Growth of ***P. aeruginosa ***on MAM supplemented with arginine only, **Plate B: **Growth of ***P. aeruginosa ***on MAM supplemented with all 22 amino acids **Plate C: **Growth of ***P. aeruginosa ***on MAM supplemented with all 22 amino acids except arginine,

The detection of specific amino acid requirements of auxotrophs revealed that 3(isolate No.1–3) had the specific requirement for arginine (Fig. 2) and 2(isolate No. 4,5) for methionine. The reproducible results were obtained on repeating the experiment thrice.

The resistance against different antibiotics was compared between auxotrophs and prototrophs. Although auxotrophs were small in number, they showed significantly higher resistance against ceftazidime (60%) as compared to prototrophs (p < 0.05). Three auxotrsophs (isolate No. 1–3) were multidrug resistant (A6 antibiogram) and the producers of ESBL. The reproducible results were obtained on repeating the experiment thrice.

### Molecular characterization of *P. aeruginosa *isolates

Molecular characterization of *P. aeruginosa *isolates (N = 350) recovered from 27 patients infected with *P. aeruginosa *was achieved by Enterobacterial Repetitive Intergenic Consensus Polymerase Chain Reaction (ERIC-PCR) and PCR-ribotyping.

### ERIC-PCR

**Figure 3:**
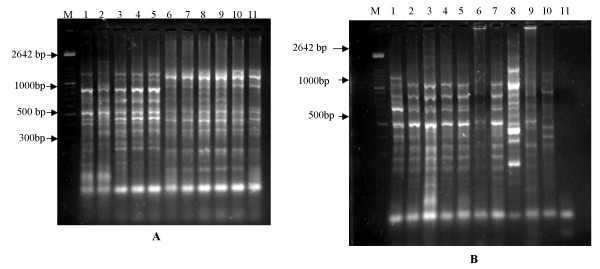
**ERIC-PCR patterns of *P. aeruginosa *isolates from the patient A. PCC. No. 180/95. **• **Lane M : **100 bp ladder, • **Lane 1 : **Pattern **E1**, • **Lane 2 : **Pattern **E3**, • **Lane 3–5 : **Pattern **E1**, • **Lane 6–11 : **Pattern **E2. B. PCC.No. 180/95 and 179/93 **• **Lane M : **100 bp ladder, • **Lane 1–7 (PCC.No.180/95) : **Pattern **E4**, • **Lane 8 (PCC. No. 179/93) : **Pattern **E5**, • **Lane 9 (PCC.No.180/95) : **Pattern **E4**, • **Lane 10 : *P. aeruginosa ***ATCC 27853 • **Lane 11 : **Negative control

A total of 33 ERIC-PCR (E1-E33) patterns were constructed (Table 3). All 27 patients infected with *P. aeruginosa *harbored different genotypes with their unique ERIC-PCR pattern except one patient (PCC.No. 499/00) who was colonized with the similar genotype (E1). Out of 8 patients who were chronically infected with *P. aeruginosa*, 4 patients (PCC.No.180/95, 179/93, 160/98 and 10/99) were colonized with 2–4 genotypes (Fig. 3A&3B). The remaining 23 patients (Group1 = 4 and Group 2 = 19 patients) were colonized with a single genotype.

### PCR-ribotyping

**Figure 4:**
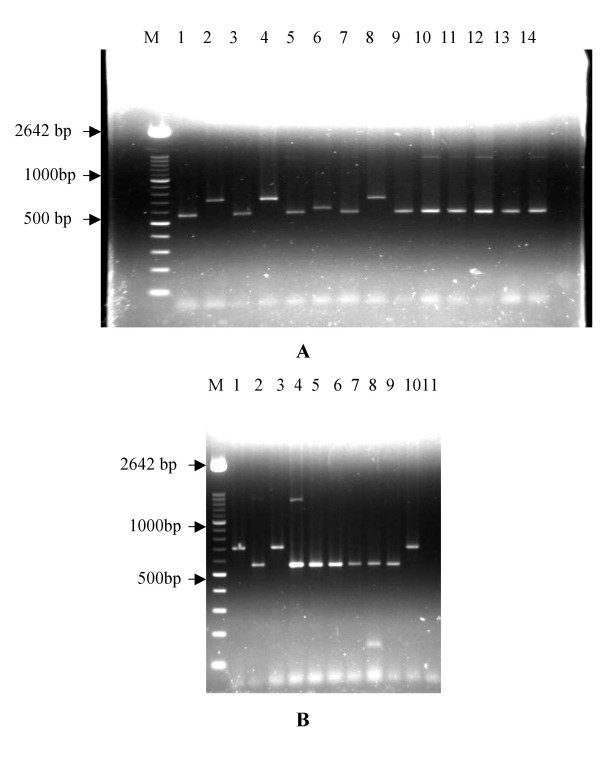
**PCR-ribotyping of *P. aeruginosa *isolates from the patient **A. PCC.No. 180/95 • **Lane M **: 100 bp ladder, • **Lane1, 3, 7 and 9 : **Pattern **P1**, • **Lane 2, 4 and 8 : **Pattern **P2**, • **Lane 5, 10–14 : **Pattern **P3**, • **Lane 6 : **Pattern **P4**B. PCC.No. 180/95 and 179/93 • **Lane M : **100 bp ladder, • **Lane 1,3 and10 (PCC.No.180/95) : **Pattern **P2**, • **Lane 2,4 (PCC.No.180/95) : **Pattern **P4**, • **Lane 5–7 and 9 (PCC.No.180/95) : **Pattern **P1**, • **Lane 8 (PCC.No.179/93 : **Pattern **P5**, • **Lane 11 : **Negative control

A total of 5 PCR ribotyping patterns (P1-P5) were obtained (Fig. 4A &4B). The molecular weight (bp) of each PCR-ribotyping pattern was P1:570, P2: 730, P3: 1500 &610, P4: 600 and P5: 570&130. Twenty four of 27 patients harbored *P. aeruginosa *with a single PCR-ribotyping Pattern P1 and 3 patients (PCC.No. 180/95, 160/98 and 179/93) were colonized with *P. aeruginosa *with 2 or more PCR-ribotyping patterns. All 3 patients were chronically infected with the organism. Isolates from patient PCC.No 180/95 Pattern P1-P4, PCC.No. 160/98 Pattern P1 and P2 and patient PCC.No. 179/93 Pattern P1 and P5.

By ERIC-PCR, *P. aeruginosa *isolates from 26/27 patients revealed different genotypes (E1-E33) while by PCR-ribotyping, 24/27 patients revealed a single PCR-ribotyping pattern (Pattern P1).

Table 3 shows the compilation of results on the patient basis including the phenotypic and genotypic characteristics of each patient.

## Discussion

Cystic fibrosis (CF) is the most common inherited fatal disease in Caucasian population. This generalized disorder of salt and water transport of exocrine glands is caused by mutations in cystic fibrosis transmembrane regulator gene in the airway epithelial cells and submucosal glands. These patients are very prone to bronchopulmonary infection due to lack of the innate defensive mechanisms. Pulmonary infection is the most common cause of morbidity and mortality in CF patients [16].

CF was thought to be extremely rare in India. However, recent reports from Indian subcontinent suggested CF to be more common in Indian population than what was previously thought [1]. In our study, we have followed all 56 patients attending the Pediatric Chest Clinic at All India Institute Of Medical Sciences, New Delhi, INDIA between August 1998-August 2001. The median age of diagnosis of patients was 4.5 years, which was much higher than what was earlier reported in the literature where the median age was found as 6 and 12 months among Caucasian American and Indian American children respectively [17]. This may be due to delayed diagnosis of CF patients in India.

*P. aeruginosa *has been the predominant bacterium associated with pulmonary infection in patients with CF [18-22]. In our study, *P. aeruginosa *was also found as an implicated organism in 82% culture positive patients and its rate of infection was significantly higher as compared to the other organisms in CF patients. The colonization with *P. aeruginosa *has been linked with pulmonary deterioration of CF patient resulting in an increased risk of hospitalization [21]. In the study, all the patients (n = 9) who required hospitalization were chronically infected with *P. aeruginosa*.

Besides morbidity, *P. aeruginosa *has been associated with increased mortality [18-22]. In the study all the 4 patients who died were chronically infected with *P. aeruginosa*. Thus significant higher rates of hospitalization and death were observed among patients chronically infected with *P. aeruginosa.*

Although prolonged and frequent administration of antibiotics has been useful in the treatment of pulmonary exacerbations, there is risk of development of multiple drug resistance in *P. aeruginosa *strains. In the study, 41% isolates were multidrug resistant and were recovered from chronically infected patients, indicating the possible role of antibiotic pressure. Similar findings have been reported elsewhere [23-25].

All three patients infected with ESBL positive isolates were found sensitive to meropenam and imipenam. The similar finding has been reported earlier where carbapenams have been found the most effective and reliable against ESBL positive isolates [26].

During chronic infection with the organism, there is progressive anatomical deterioration of the CF lung, which provides a highly spatial structured environment leading to diversification of the organism into six different colony morphological types-Type1-Type 6[12]. This diversification enhances the capacity of an organism to survive in lower respiratory tract of CF patients [13]. In the present study, 7/8(87.5%) chronically infected patients harbored different colony morphological types in the sputum samples.

Once *P. aeruginosa *has colonized the respiratory passages of CF patients, it leads to the induction of mucoidity, resulting in persistent infections. In the study, 6/8(75%) patients who were chronically infected with *P. aeruginosa *harbored mucoid *P. aeruginosa *in their respiratory samples. Our finding in agreement with the earlier reports where an increased association of mucoidity with chronicity of infection was observed [4,27,28]. Out of 4 patients who died, 3(75%) harbored mucoid colonies in their respiratory samples signifying the association of mucoidy with high mortality in CF patients. Our finding was in accordance with the earlier study where association of mucoidity with high risk of death was observed [26].

The antimicrobial susceptibility among mucoid and nonmucoid isolates was compared. The mucoid isolates showed increased resistance against gentamicin and lower resistance against beta lactam (7.4% to piperacillin and no resistance to ceftazidime). The results were analogy to the studies where high resistance to aminoglycosides [29,30] and low resistance to beta lactam [31,32] was observed among mucoid isolates.

With chronicity of infection, there is an increase in concentration of amino acids in bronchial secretions, which is due to active epithelial transport, plasma exudation and protease activity in lung tissue and surface fluid. This results in development of auxotrophy for certain amino acids required for growth. In the present study, auxotrophic *P. aeruginosa *was found in 2/27(7.4%) patients in contrary to an earlier report where auxotrophy of *P. aeruginosa *was found in 86% patients infected with the organism [33]. The reason for low percentage of auxotrophs among our patients may be due to lower number of chronic patients in this study.

Furthermore in the study, 3/5 auxotrophs had arginine requirement and the remaining 2 were auxotrophic for methionine. This was in agreement with the earlier reports where methionine and arginine were considered as the most commonly required amino acids by auxotrophs [14,33,34]. As these strains could be more resistant to antibiotics, it is important to study auxotrophy of isolates in CF patients with severe *P. aeruginosa *infection in order to plan out appropriate therapy for the management of CF patients.

Bacterial typing schemes are based on phenotypic or genotypic analysis of multiple isolates within a particular species. Such analysis helps in investigation of outbreaks and determines the recurrence or relapse of an infection. The epidemiology of *P. aeruginosa *infection in CF patients has been hampered since the conventional phenotyping methods-phage and pyocin typing have poor discriminatory power and poor reproducibility respectively. Genetic typing methods have shown to be more discriminatory than phenotypic methods for typing *P. aeruginosa *CF isolates [35]. Various genotypic typing methods- restriction fragment length polymorphism (RFLP) analysis of *tox A I *and *pil *A loci, pulse field gel electrophoresis (PFGE), random amplified DNA polymorphism (RAPD) PCR have been used to identify the number of strains colonizing the CF patient [13].

PCR-ribotyping is based on the uniform spacer region in rRNA operons of *P. aeruginosa *[36] resulting in low degree of heterogeneity of rRNA operons. In the study, majority of isolates showed the single PCR-ribotyping Pattern P1, probably due to the above reason. Hence for the study of dynamic of *P. aeruginosa *strain variation, ERIC-PCR may be a useful tool.

By ERIC-PCR, all 27 culture positive patients except one were found colonized with the unique genotype (E1-E33). This indicates that cross-colonization among unrelated CF patients was uncommon. The results were concordant to the earlier studies where cross-infection between unrelated patients was considered unusual [37,38]. All 27 patients except 4 were colonized with the single persistent genotype. These 4 patients were chronically infected and were colonized with 2 or more genotypes. Our results were in agreement with the previous studies where majority of CF patients were colonized with a predominant genotype for longer period of time but high degree of genotypic variability within patients have been observed suggesting that the colonizing strain may occasionally be replaced [13,39-41].

Thus we have found that CF is a problem in our country but delayed diagnosis and low index of suspicion have delayed the appropriate management of these patients. Although all the patients attending the Pediatric chest clinic were included in the study, the number of patients, especially the chronic ones, was limited in India, which further limited the number of chronic patients. Although the small number of patients in our study is not sufficient to answer all the epidemiological questions, these results indicate that a larger scale CF study should be conducted in India.

## Conclusion

*P. aeruginosa *is the major colonizer of the respiratory passages of CF patients. The patients with chronic infection showed significantly higher rates of hospitalization and death. With chronicity of infection, the organism undergoes phenotypic and genotypic variations. Among phenotypic variations, the organism becomes multidrug resistant, diversifies its colony morphology, acquires mucoidity and becomes auxotrophic for various amino acids. The multiple genotypes have been found to colonize the respiratory passages of CF patients.

Thus in a particular clinical set up, high index of suspicion should be there for diagnosis of CF patients so as to prevent the delay in diagnosis and management of CF patients. The routine microbiological examination of respiratory samples from CF patients are advised to study the characteristics of the infecting agents of which *P. aeruginosa *is the most common.

## Methods

### Subjects

All patients with CF (age equal to or less than 15 years) attending the Pediatric Chest Clinic at All India Institute Of Medical Sciences between August 1998-August 2001 were included in the study. There was a regular monthly follow-up of each patient to the clinic. Patients who required hospitalization were admitted to the Pediatric ward and were followed daily. The diagnosis of CF was based on both clinical and laboratory parameters [42]. For analysis, patients were divided into 2 groups -Group 1 and Group 2 based on the chronicity of infection with *P. aeruginosa *[12]. Patients under Group 1 were chronically infected with *P. aeruginosa *for the past two years and Group 2 included patients who were intermittently colonized with the organism.

### Sample collection

During each visit of a patient to the clinic and during the period of hospitalization, a representative sample – sputum and/or throat swab after physiotherapy was collected [41]. The sputum sample was collected directly in a sterile vial after coughing. Throat swab was considered as an alternative respiratory sample and was collected from children who were either below 2 years of age or were not able to expectorate sputum [43].

### Isolation and characterization of *P. aeruginosa *strains

The sputum was vortexed (for complete homogenization) and cultured on Chocolate agar, Blood agar containing 5% sheep blood and Mac Conkey agar (Hi Media, Mumbai). The plates were incubated at 37°C for 18 hrs. *P. aeruginosa *was identified by standard bacteriological methods [44] and its different colony morphotypes were recorded [12].

### Antibiotic susceptibility

#### Antibiogram

The single representative from each colony morphotype was taken and tested for antimicrobial susceptibility by agar dilution method as per NCCLS guidelines [45]. Of the six colony morphotypes, Type 5 was the mucoid morphotype and the rest morphotypes(Type 1–4 and Type 6) were the nonmucoid morphotypes. Depending on antimicrobial susceptibility of an organism, antibiograms were constructed where intermediate and resistant categories were clubbed. The antibiotics tested were gentamicin, ciprofloxacin, piperacillin and ceftazidime. All these antibiotics were procured from Hi Media, Mumbai. *P. aeruginosa *ATCC 27853 and *Escherchia coli *ATCC 25922(ATCC, USA) served as the control strains. Those isolates, which showed resistance to two or more than two antibiotics were considered multidrug resistant [10].

### Production of Extended spectrum beta lactamase (ESBL)

Those isolates, which showed resistance to ceftazidime (CAZ) were tested for ESBL production by Disc Diffusion according to NCCLS guidelines [46] and by Etest method (AB BIODISK, Sweden). All ESBL positive isolates were further tested for carbapenam-imipenam and carbapenam susceptibilty by disk diffusion method [46].

### Phenotypic characterization of *P. aeruginosa *isolates

Different phenotypic features of *P. aeruginosa *were studied in CF patients. The phenotypic features included colony morphotypes and auxotrophy.

### Colony morphotypes

Based on colony morphology of the organism, there are six different colony morphotypes of *P. aeruginosa *[12]. The presence of six different morphotypes was observed in respiratory samples of CF patients. Type 5 morphotype showed mucoidy, which is one of the virulence factor of the organism.

### Auxotrophy

All *P. aeruginosa *isolates from CF patients were tested for auxotrophy. The inoculum was adjusted to 0.5 Mc Farland and diluted to have the final concentration of 10^4^cells/μl. One microlitre of the inoculum was spotted on Mueller Hinton agar (MHA) and Minimal agar medium (MAM) where MHA served as the complex medium and MAM as nutrient deficient medium. The plates were incubated at 37°C for 48 hrs. Those isolates, which did not grow on MAM plate but grew on MHA were considered auxotrophs. Those isolates, which were able to grow on both MHA and MAM plates were considered prototrophs. *P. aeruginosa *NCTC 50184 (*meth *^-^) and *P. aeruginosa *ATCC 27853 were included as the known auxotrophic and prototrophic strain respectively. The specific amino acid requirements were identified.

#### Identification of specific amino acid requirements

The stock solution of all 23 L-amino acids including cysteine, proline, lysine, arginine (Sigma, USA), cystine, methionine, serine (CDH), glutamine, glutamic acid, glycine, histidine, hydroxy-proline, isoleucine, leucine, ornithine, phenylalanine, alanine, asparagine, aspartic acid, threonine, tryptophan, tyrosine and valine (HiMedia, Mumbai) were prepared at the concentration of 2 mg/ml in either sterile distilled water or the appropriate solvent. All the amino acids were filter sterilized by 0.45 μm Millipore filter (Millipore, USA). The specific amino acid/acids requirement was determined by two methods [34].

The first method helps in detection of single or multiple amino acids. In this method, a single amino acid at the concentration of 20 μg/ml was added to each MAM plate. The plates were inoculated and incubated at 37°C for 48 hrs. The growth on specific amino acid containing plates indicated the requirement for single/multiple amino acids while no growth in any of the MAM plate supplemented with amino acid indicated either the requirement for two or more amino acids in combination or some other growth factor.

The second method helps in detection of combination of amino acids. All amino acids (at the concentration of 20 μg/ml) less one were added to molten MAM. The plates were inoculated and incubated as described above. The specific requirement for an amino acid/combination of amino acids was evident, if the isolate failed to grow on agar not containing that particular amino acid.

#### Antibiotic resistance

The antibiotic susceptibility of auxotrophs and prototrophs was determined by agar dilution method and the antibiotic resistance was compared between them. The antibiotics tested were gentamicin, ciprofloxacin, piperacillin and ceftazidime.

### Molecular characterization of *P. aeruginosa *isolates

Molecular typing of *P. aeruginosa *isolates from all culture positive patients was done by ERIC-PCR and PCR-ribotyping. Both the molecular methods were carried out as described earlier [12].

### Statistical analysis

To see the association between the categorical variables, Chi square test (with Yates correction)/Fischer's exact test was used. The result was considered significant at 5% level of significance (p < 0.05). SAS 8.0 statistical package was used for analysis.

## Authors' contributions

GA: Technical procedures, study design and manuscript preparation.

AK: Infrastructure, time-to-time guidance and participated in microbiological experiments.

BKD: Involved in the molecular studies.

SKK: Helped in collection of the samples. Provided case histories of CF patients and helped in evaluation of clinical data.

SND: Performed the statistical analysis.

## Figures and Tables

**Table 1:** Antibiograms

**Antibiogram**	**Susceptibility to antibiotics**	**Total isolates (%)**
A1	S to all	102(29.1)
A2	R to Genta only	81(23.1)
A3	R to Genta+Cipro	85(24.3)
A4	R to Cipro only	24(6.9)
A5	R to Genta+Pipra	23(6.6)
A6	R to Genta+Cipro+CAZ	24(6.9)
A7	R to Genta+CAZ	5(1.4)
A8	R to Genta+Cipro+Pipra	6(1.7)

Genta: Gentamicin; Cipro: Ciprofloxacin; Pipra: Piperacillin; CAZ: Ceftazidime CAZ:

S: Sensitive; R: Resistant

+: Indicates "and"

**Table 2:** Distribution of eight antibiograms (A1-A8) among six colony morphological types of *P. aeruginosa*.

**Colony Morphotype (N)**	**Total Number of isolate (%) showing respective Antibiograms**							
	
	**A1**	**A2**	**A3**	**A4**	**A5**	**A6**	**A7**	**A8**
Type 1 (194)	102 (52.5)	23 (11.8)	27 (10.3)	4 (1.5)	15 (7.7)	14 (7.2)	3 (1.5)	6(3)
Type2 (26)	5 (19.2)	0	10(38.5)	2(7.7)	1(3.8)	7(27)	1(3.8)	0
Type3 (23)	9 (39.1)	0	6(26.1)	5(21.7)	0	3(13.0)	0	0
Type4 (5)	2 (40)	0	2 (40)	1(20)	0	0	0	0
Type5 (94)	0	39 (41.5)	48 (51.1)	0	7(7.4)	0	0	0
Type6 (8)	0	5 (62.5)	1 (12.5)	0	0	1(12.5)	0	1(12.5)

N = Total Number of isolates

**Table 3:** Phenotypic and genotypic characteristics of *P. aeruginosa *isolated from cystic fibrosis patients (Total No. of patients infected with *P. aeruginosa *= 27)

**S.No.**	**PCC.No.**	**Total isolates**	**Phenotypic features**				**Genotypic pattern**	
			**Colony morphotype**	**Antibiogram**	**ESBL**	**Auxotrophy**	**ERIC-PCR**	**PCR-ribotyping**

**Group1**								
1	180/95	146	Type 1–6	A1-A4, A6-A8	+	+	E1-E4	P1-P4
2	179/93	59	Type 1,2,5	A1-A5, A7	-	-	E5-E7	P1, P5
3	174/97	22	Type 1	A1	-	-	E8	P1
4	308/98	22	Type1–3, 5	A1, A2, A5	-	-	E9	P1
5	10/99	22	Type1, 3	A1, A2, A4, A5	-	-	E10, E11	P1
6	160/98	19	Type1, 3,5	A3-A5, A7, A8	+	-	E12, E13	P1, P2
7	121/99	19	Type1–3, 5	A1, A2	-	-	E14	P1
8	308/00	4	Type 2,3,5	A1, A4	-	-	E15	P1
**Group2**								
9	167/98	4	Type1	A1	-	-	E16	P1
10	250/99	4	Type1	A1	-	-	E17	P1
11	5/00	4	Type1	A1	-	-	E18	P1
12	256/99	3	Type1	A1, A2	-	-	E19	P1
13	337/98	3	Type1	A1	-	-	E20	P1
14	38/95	2	Type1	A1	-	-	E21	P1
15	202/98	2	Type1	A1, A3	-	-	E22	P1
16	312/99	2	Type1	A1	-	-	E23	P1
17	170/99	2	Type1	A1	-	-	E24	P1
18	499/00	2	Type1	A6	+	+	E1	P1
19	302/96	1	Type1	A1	-	-	E25	P1
20	95/96	1	Type1	A1	-	-	E26	P1
21	82/96	1	Type1	A1	-	-	E27	P1
22	8/97	1	Type1	A1	-	-	E28	P1
23	203/99	1	Type1	A1	-	-	E29	P1
24	133/99	1	Type1	A1	-	-	E30	P1
25	124/00	1	Type1	A1	-	-	E31	P1
26	452/00	1	Type1	A1	-	-	E32	P1
27	76/01	1	Type1	A1	-	-	E33	P1

Genta: Gentamicin; Cipro: Ciprofloxacin; Pipra: Piperacillin; CAZ: Ceftazidime CAZ: S: Sensitive; R: Resistant +: Indicates "and"
